# RIPK3 in necroptosis and cancer

**DOI:** 10.1016/j.mocell.2025.100199

**Published:** 2025-02-24

**Authors:** Michael J. Morgan, You-Sun Kim

**Affiliations:** 1 Department of Natural Sciences, Northeastern State University, Tahlequah, OK 74464, USA; 2 Department of Biochemistry, Ajou University School of Medicine, Ajou University, Suwon 16499, Korea; 3 Department of Biomedical Sciences, Graduate School, Ajou University, Suwon 16499, Korea

**Keywords:** Cancer cell death, Damage-associated molecular patterns, MLKL, Necroptosis, Receptor-interacting protein kinase-3

## Abstract

Receptor-interacting protein kinase-3 is essential for the cell death pathway called necroptosis. Necroptosis is activated by the death receptor ligands and pattern recognition receptors of the innate immune system, leading to significant consequences in inflammation and in diseases, particularly cancer. Necroptosis is highly proinflammatory compared with other modes of cell death because cell membrane integrity is lost, resulting in releases of cytokines and damage-associated molecular patterns that potentiate inflammation and activate the immune system. We discuss various ways that necroptosis is triggered along with its potential role in cancer and therapy.

## INTRODUCTION

Programmed cell death is a genetically encoded process by which cells are deleted in response to physiological and pathological signals that typically occur during development, tissue homeostasis, damage, infection, or neoplastic threat (Galluzzi et al., 2018). External stimuli (eg, chemotherapy) may also trigger a similar “regulated cell death” process via similar genetically encoded means (Galluzzi et al., 2018). While such cell death processes occur through a number of mechanisms, the process of apoptosis is often considered the primary or most physiological mode of cell death and was the first genetically encoded cell death process to be discovered. Downstream execution of apoptosis occurs via caspase family cysteine proteases that cleave target proteins involved in cellular structure or essential cell processes. For instance, cytoskeletal proteins, survival factors, DNA repair enzymes, cell cycle regulators, and transcription factors are cleaved, while some caspases cut and activate enzymes that target DNA itself. Destruction of the cell via apoptosis is a very orderly process characterized by shrinkage of the cell and its organelles, as well as nuclear and chromatic condensation, DNA fragmentation, mitochondrial permeability, and membrane extensions that separate and form around parts of cytoplasm and organelles. These then condense into apoptotic bodies that can be rapidly phagocytosed by surrounding cells and/or immune system phagocytes. Intracellular contents are, therefore, not released into the surrounding tissues during apoptosis, thus preventing inflammation.

While apoptosis is often the primary mode of cell death, numerous alternative cell death pathways have now been discovered that occur in the absence of caspase activation. A second major form of programmed death was discovered just before the turn of the century, originally called “programmed necrotic cell death” or “programmed necrosis” (Denecker et al., 2001, Grooten et al., 1993, Holler et al., 2000, Laster et al., 1988, Vercammen et al., 1998). It was named so because it proceeded with a more “necrotic-like” morphology and is now referred to as “necroptosis” (Degterev et al., 2005). Unlike apoptosis, necroptotic cell death is due largely to direct permeabilization of the plasma membrane. Cellular factors that leak through the membrane, therefore, promote inflammation and immune cell activation (Fiers et al., 1999, Kroemer et al., 2005). Apoptosis, on the other hand, only activates the immune system occasionally and is considered far less proinflammatory.

Necroptosis was originally discovered as “not being the same as apoptosis.” That is, it was caspase-independent; now, many other death pathways are known to be either caspase-independent or different in the way that caspases are activated or the way that cell death occurs. Receptor-interacting protein kinase-1 was the first factor found that, when deleted or silenced, prevented TNF-α-mediated necroptosis (Holler et al., 2000, Lin et al., 2004). This protein turned out to be one of several that could directly activate its sister kinase RIPK3 (Cho et al., 2009, Smith et al., 2009, Zhang et al., 2009), which is the essential kinase required (by current definition) for this form of death. The RIPK3 protein consists of 2 domains: a C-terminal domain by which it is activated, and an N-terminal kinase domain, which is required for initiating necroptosis via phosphorylation of a pseudokinase called mixed lineage kinase domain-like protein (MLKL) (Murphy et al., 2013, Sun et al., 2012, Wu et al., 2013, Zhao et al., 2012). The N-terminal 4-/5-helical bundle domain of MLKL in its inactivated state is firmly associated with its pseudokinase domain, but RIPK3-mediated phosphorylation of MLKL releases this interaction causing MLKL oligomerization (Hildebrand et al., 2014, Murphy et al., 2013, Petrie et al., 2018). Upon oligomerization, MLKL translocates to the plasma membrane where it has been reported to bind to phosphoinositides (Dondelinger et al., 2014, Dovey et al., 2018, Quarato et al., 2016, Rosenfeld et al., 2014). It then causes membrane permeability through a less understood mechanism, perhaps through the formation of channels or pores (Huang et al., 2017, Ros et al., 2017, Xia et al., 2016) or indirectly through interaction with ion channels that let various cations through (Cai et al., 2014, Chen et al., 2014). In some cases, modification of MLKL by other factors, including ubiquitylation of MLKL, can also be necessary for necroptosis to occur (Petrie et al., 2020, Samson et al., 2020). MLKL may also have other functions and may also be transported or positively or negatively regulated by ESCRT or other proteins (Fan et al., 2019, Garcia et al., 2021, Gong et al., 2017b, Liu et al., 2021).

### Activation of Necroptosis

**Fig. 1:**
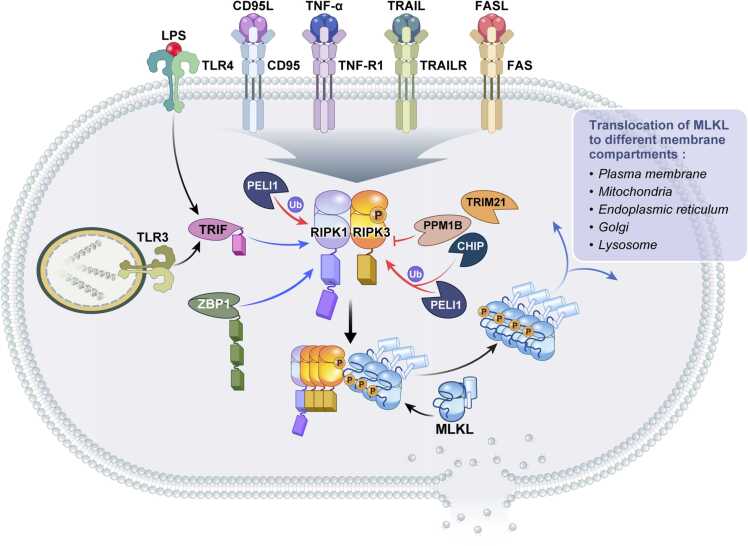
Activation of RIPK3 by multiple stimuli. RIPK3 can be activated via various receptors when bound by their respective ligands. These are TNF receptor 1 (TNF-R1), TRAIL receptor, Toll-like receptors (TLR3/4), CD95, and FAS. In the first 3 of these pathways (but not TLR3/4 or ZBP1), RIPK1 is required and binds to RIPK3 through its receptor-interacting protein homotypic interaction motif (RHIM). In the case of Z-DNA-binding protein-1 (ZBP1)/DAI, RIPK3 is recruited directly via the ZBP1 RHIM domain, while in the case of TLR3/4, RIPK3 is recruited indirectly via the RHIM domain of TRIF. Once activated, RIPK3 autophosphorylates and then phosphorylates and activates MLKL to induce a conformational change and translocation to the membrane, where membrane permeabilization follows. During this process, post-translational modifications positively and negatively regulate the necroptosis pathway. Two E3 ligases, Pellino-1 (PELI1) and carboxy terminus of HSC70-interacting protein (CHIP), may control the basal threshold of necroptosis. Another E3 ubiquitin ligase, TRIM21, is proposed to be a regulator of necroptotic cell death in response to TRAIL. Protein phosphatase 1B suppresses necroptosis by dephosphorylating RIPK3.

Necroptosis is activated by signals initiated by death receptors via their death receptor ligands, TNF-α, FasL, and TRAIL (Holler et al., 2000, Lin et al., 2004, Vandenabeele et al., 2010b, Vanlangenakker et al., 2012), as well as pattern recognition receptors (PRRs) of the innate immune system that recognize cell stress, such as Toll-like receptors TLR3 and TLR4, and ZBP1/DAI (He et al., 2011, Jiao et al., 2020, Kaiser et al., 2013a, Maelfait et al., 2017, Nogusa et al., 2016, Upton et al., 2012) (Fig. 1). Each of these pathways requires a receptor-interacting protein homotypic interaction motif (RHIM) protein that recruits the C-terminal RHIM domain of RIPK3, resulting in RIPK3 oligomerization and thus activating RIPK3 kinase activity in what is termed the necrosome (Kaiser et al., 2013a, Sun et al., 2002). RIPK1, the death domain (DD)-containing sister kinase of RIPK3, is the RHIM-containing adapter in death receptor signaling that recruits RIPK3 in response to all 3 aforementioned ligands (Sun et al., 2002). The adapter Toll/IL-1 receptor domain-containing adapter protein inducing interferon-β (TICAM-1, more commonly referred to as TRIF) serves as the RHIM-containing adapter for both TLR3 and TLR4 pathways, which respond to double-stranded RNA ligands and lipopolysaccharide, respectively (Tummers and Green, 2022). ZBP1/DAI is a cytoplasmic receptor that senses Z (left-handed) double-stranded (ds) DNA and RNA. ZBP1 is an RHIM-containing protein that directly recruits RIPK3 rather than through an adapter (He et al., 2011, Kaiser et al., 2013a, Upton et al., 2012). Necroptosis can also be induced indirectly through other PRRs via upregulation of these pathways via transcription (Brault et al., 2018, Robinson et al., 2012, Schock et al., 2017). PRRs that can induce indirect necroptosis in this manner include interferon receptor INFAR1, retinoic acid-inducible gene I (RIG-I/DDX58), and STING1 (Brault et al., 2018, Robinson et al., 2012, Schock et al., 2017).

RIPK3, as mentioned, is recruited to TLR4 via TRIF adapter recruitment to the TLR4 protein. This may also recruit RIPK1 secondarily but is not dependent on RIPK1 (He et al., 2011, Kaiser et al., 2013a). Myeloid differentiation primary response protein 88 (MYD88) is also recruited to TLR4 and can also cause the RIPK1-dependent activation of necrosome, though it is perhaps indirectly regulated via gene upregulation of the TNF/TNFRI pathway (Ariana et al., 2020, Kaiser et al., 2013a, McComb et al., 2014). The cIAPs 1 and 2 inhibit the necroptosis induced through TLR4 in a similar manner as they do the TNF pathway (McComb et al., 2012).

TLR3 is an intracellular PRR that also mediates necroptosis via TRIF (He et al., 2011, Kaiser et al., 2013a, Upton et al., 2012) but is activated in response to double-stranded (ds)RNA, such as poly(I:C), or UVB-damaged self-RNA (Bernard et al., 2012). TLR3-dependent necroptosis does not generally require RIPK1 in most cells, but macrophages may require RIPK1 for TLR3-mediated necroptosis (Kaiser et al., 2013a).

ZBP1/DAI is also a cytoplasmic PRR that activates both necroptosis and apoptosis (Jiao et al., 2020, Maelfait et al., 2017, Nogusa et al., 2016, Upton et al., 2012) in response to pathogens zDNA and zRNA, sensing these via 2 N-terminal Z nucleotide-binding sites (Schwartz et al., 2001). ZBP1 does not need an adapter protein and, as mentioned recruits RIPK3 via its own C-terminal RHIM domain (Upton et al., 2012), which then activates MLKL to trigger necroptosis (Nogusa et al., 2016). RIPK1 is not essential for ZBP1-induced necroptosis, and its presence actually inhibits both ZBP1-mediated apoptosis and necroptosis instead (Klionsky et al., 2016, Newton et al., 2016, Solon et al., 2024).

During death receptor signaling, RIPK3 is brought to receptor complexes via RIPK1, which is essential for necroptosis in this case (Chan et al., 2003, Degterev et al., 2008, Holler et al., 2000). RIPK1, in turn, has a C-terminal DD that is recruited to the receptor signaling receptor complexes by binding similar DDs contained within the receptor intracellular domains and/or in adapter proteins (Morgan and Liu, 2010, Vandenabeele et al., 2010b). In the TNF-α pathway mediated via TNFRI (TNFRSF1B), the adapter proteins TRADD and RIPK1 bind together to the TNF-R1 receptor upon treatment with TNF-α (Morgan and Liu, 2010, Vandenabeele et al., 2010b). RIPK1 is then modified by several post-translational modifications, including phosphorylation and polyubiquitination (Cockram et al., 2021), through several mechanisms (see below).

K63-directed ubiquitination of TRADD increases its affinity for TRAF2, which is recruited and then subsequently recruits cellular inhibitors of apoptosis protein-1 (cIAP1) and cIAP2, which are E3 ligases. These modify RIPK1 on its internal domain with K63-linked ubiquitination or (cIAP1-mediated) K48-linked chains (Cockram et al., 2021). Further recruitment of the linear ubiquitin chain assembly complex promotes linear M1 polyubiquitination (Iwai, 2021). Polyubiquitination of RIPK1 by K63-linked chains allows the recruitment of the IκB kinases and TGFβ-activated kinase-1 (TAK1) to the complexes, which activate ERK, JNK, and p38 Map kinases, as well as nuclear factor κB (NF-κB) (Seo et al., 2021). M1 polyubiquitination also promotes IκB kinases subunit recruitment (Cockram et al., 2021). Ubiquitin hydrolases CYLD (Wright et al., 2007), A20 (Wertz et al., 2004), and OTULIN (Heger et al., 2018) can remove the K63-linked or linear ubiquitination modifications of RIPK1, promoting different interactions for RIPK1. CYLD activation in the receptor complex tends to promote necroptosis by allowing secondary complexes and RIPK1/RIPK3 interactions to occur (Moquin et al., 2013). In contrast, K48-linked ubiquitination promotes proteasomal degradation of RIPK3, which prevents it from binding to RIPK3 and activating necroptosis and also causes its degradation.

A secondary complex containing TRADD/RIPK1 and FADD/caspase-8 forms away from the receptor to which RIPK3 is secondarily recruited (Han et al., 2011, Vandenabeele et al., 2010a). This complex termed the necrosome, then regulates apoptosis as well as necroptosis. The amount of caspase-8 and RIPK3 in the complex appears to determine which, as high amounts of caspase-8 trigger apoptosis and cleave RIPK3, while low caspase-8 activity/high RIPK3 activity leads to necroptosis.

The RIPK1-RIPK3 interaction is contingent on the kinase activity of both kinases (Cho et al., 2009, Degterev et al., 2008). The kinases autophosphorylate themselves but do not phosphorylate each other (Petrie et al., 2019). The resulting necroptotic complex assembles in an amyloid fiber (Chen et al., 2022, Li et al., 2012), where RIPK3 phosphorylates MLKL to lead to necroptotic cell death (Sun et al., 2012, Zhao et al., 2012), (Murphy et al., 2013, Wu et al., 2013). Curiously, RIPK1 not only positively regulates the activity of the necrosome complex in the TNF pathway but also keeps constitutive RIPK3 activity in check, preventing premature necrotic cell death (Dannappel et al., 2014, Dillon et al., 2014, Orozco et al., 2014, Rickard et al., 2014).

Protein phosphatase 1B suppresses necroptosis by dephosphorylating RIPK3, which then prevents MLKL from staying in the necrosome (Chen et al., 2015). Casein kinase family members phosphorylate on RIPK3 serine 227 (Hanna-Addams et al., 2020, Lee et al., 2019), the same phosphorylation that occurs via autophosphorylation. This keeps RIPK3 active in the complex.

In contrast to events that occur in the receptor complex, 2 different K63 ubiquitination modifications of RIPK1 in secondary complexes promote necrosome formation. K63-linked polyubiquitination of RIPK1 by c-Cbl promotes a detergent-insoluble aggregation of RIPK1 when TAK1 is inhibited, thus increasing necroptosis (Amin et al., 2018). Pellino-1-mediated K63-linked modification of kinase-active RIPK1 increases affinity for RIPK3 (Wang et al., 2017).

Conversely, HSC70-interacting protein (CHIP) ubiquitination on the C-terminus of inactivated cytosolic RIPK1 targets it for lysosomal degradation (Seo et al., 2016). CHIP also targets RIPK3 in a similar manner (Seo et al., 2016). Pellino-1, on the other hand, mediates K48 polyubiquitination of activated RIPK3 in the necrosome, thus causing proteasomal degradation (Choi et al., 2018, Morgan and Kim, 2015a). These 2 E3 ligases likely determine basal necroptotic signaling in the cell.

The mechanism of Fas receptor (FAS)-induced necroptosis is believed to be similar to that induced through TNF-R1, except that FADD and RIPK1 are recruited directly to the FAS cytoplasmic DD and caspase-8 is also then recruited into the receptor signaling complex (Morgan and Liu, 2013). FADD is additionally required for FAS-induced necroptosis; FADD-deficient cells are highly resistant to FAS-mediated necroptosis (and apoptosis) (Holler et al., 2000, Lawrence and Chow, 2005, Yeh et al., 1998). As in the TNF pathway, caspase-8 and cIAP inhibition promotes the switch from apoptosis to necroptosis (Bohgaki et al., 2011, Geserick et al., 2009, Holler et al., 2000, Kaiser et al., 2011, Oberst et al., 2011, Vanlangenakker et al., 2011).

TRAIL-induced necroptosis is mediated via DR4 and DR5 receptors similarly to the other death receptors with RIPK1 being required (Fullsack et al., 2019, Jouan-Lanhouet et al., 2012, Meurette et al., 2007). Likewise, necroptosis is potentiated by this pathway when apoptosis is blocked, when TAK1 is deficient (Dondelinger et al., 2013, Goodall et al., 2016, Morioka et al., 2009), or when cIAPs are inhibited (Dondelinger et al., 2013, Smith et al., 2009). FADD is also essential (Fullsack et al., 2019, Kuang et al., 2000, Peter, 2000, Sprick et al., 2000), as RIPK1 does not bind directly to the receptors, and is instead recruited by FADD and caspase-8 (Cao et al., 2011, Henry and Martin, 2017, Kim et al., 2011). As in TNF signaling, linear ubiquitination of RIPK1 by linear ubiquitin chain assembly complex blocks TRAIL-induced necroptosis (Lafont et al., 2017). The E3 ubiquitin ligase TRIM21, however, is an upregulator of TRAIL-induced necroptosis (Simoes Eugenio et al., 2021).

Of course, most of these necroptosis signaling pathways above are triggered by infectious pathogens. One hypothesis is that necroptosis is a secondary evolved mechanism that (1) eliminates infected cells, thus preventing viral replication, and (2) induces inflammatory responses to respond to pathogens, but may also lead to termination of long-term inflammation in some cases (Kitur et al., 2016). Two important facts that argue for these hypotheses are that animals that are RIPK3-deficient are highly sensitive to certain types of viral infections and that some pathogens have evolved mechanisms to suppress necroptosis (Nailwal and Chan, 2019, Ye et al., 2023). Pathogens have also evolved mechanisms to avoid apoptosis, by inhibiting the activity of specific caspases, for instance (Faherty and Maurelli, 2008, Kaiser et al., 2013b), which may then trigger necroptosis.

Importantly, as apoptosis is considered the dominant pathway when it is activated, it inactivates necroptosis via various mechanisms, many of which involve cleavage of the RHIM-containing adapters and preventing RIPK3 recruitment/activation. RIPK1 is cleaved by caspase-8 (Lin et al., 1999), which in death receptor pathways, is present in the necrosome complex, along with FADD. CYLD is also cleaved by caspase-8 during apoptosis as RIPK1 is thus reducing death receptor necroptosis (O'Donnell et al., 2011). Like RIPK1, TRIF is cleaved by active caspases, including caspase-8, allowing apoptosis to downregulate necroptosis induced by TLR3 and TLR4. ZBP1 inactivation by apoptosis is more complicated due to it often being activated during what is termed PANoptosis, in which necroptosis, apoptosis, and pyroptosis are all superimposed among each other. However, it is clear that ZBP1 deletion protects from necroptosis occurring in caspase-8/TNF-R1 KO mice (Solon et al., 2024) or RIPK1 and caspase-8 mutations together (Newton et al., 2016), inferring that it is also inhibited by caspase cleavage. In knockout mice, apoptotic inactivation of necroptosis is evident. Gene deletions of apoptotic pathway proteins are rescued completely or partially by RIPK1/RIPK3 deficiency, including caspase-8 knockout, FADD knockout, cFLIP-FADD double knockout (but not cFLIP knockout), XIAP-cIAP1 double knockout, and cIAP1-cIAP2 double knockout (Bonnet et al., 2011, Ch'en et al., 2011, Dillon et al., 2012, Kaiser et al., 2011, Lu et al., 2011, Moulin et al., 2012, Oberst et al., 2011, Zhang et al., 2011).

### RIPK3 and Necroptosis in Cancer

The roles of RIPK3 in cancer, in many cases, seem paradoxical. There are some data that have suggested that RIPK3 and necroptosis-mediated inflammation contributes to tumorigenesis through an inflammatory and immunosuppressive tumor microenvironment (Jiao et al., 2018, Seifert et al., 2016, Strilic et al., 2016, Vucur et al., 2023, Wang et al., 2018, Zhang et al., 2023, Zhong et al., 2023). We will discuss this presently, but conversely, many investigations have concluded that necroptosis and/or RIPK3 had a role in cancer mitigation and control (Cho et al., 2024, Colbert et al., 2013, Conev et al., 2019, Ertao et al., 2016, Koo et al., 2015, Morgan and Kim, 2015b, Murphy et al., 2013, Park et al., 2009, Seifert et al., 2016). For example, a recent study of ours concluded that RIPK3 KO mice were more sensitive to developing long-term lymphomas, especially when coupled with p53 loss (Hwang et al., 2022). Although RIPK3 is expressed in normal primary cells and tissues (Kasof et al., 2000, Koo et al., 2015, Newton et al., 2004, Sun et al., 1999, Yang et al., 2005, Yu et al., 1999), we observed a few years ago that RIPK3 expression is almost completely silenced (both at the mRNA and protein level) in a large majority of cancer cell lines from a large variety of different tissues, as well as many primary cancers. This result was similar to what was observed by Kasof et al. (2000) earlier in a more limited study. We determined that this phenomenon was due to methylation of its promoter (Koo et al., 2015), though hematopoietic compartments were less likely to have complete RIPK3 silencing (20%). This was reversible, as treatment with hypomethylating agents decitabine and azacytidine reduced DNA methylation and restored both RIPK3 mRNA and protein expression (Koo et al., 2015), meaning that it could potentially be therapeutically possible to restore RIPK3 expression.

A number of studies have shown that higher RIPK3, MLKL expression, and necroptosis signatures are often positively correlated with better prognoses, anticancer effects, and responses to therapy (Cho et al., 2024, Colbert et al., 2013, Conev et al., 2019, Ertao et al., 2016, Feng et al., 2015, Koo et al., 2015, Liang et al., 2024, Lin et al., 2023, Mannion et al., 2024, Nicole et al., 2022) This, along with the selection against RIPK3 expression in cancer (Kasof et al., 2000, Koo et al., 2015), raises the important question of why RIPK3 expression is typically low in cancer and why this low expression is associated with better patient outcomes. Several possibilities are suggested by what we know about RIPK3 and necroptosis. Some are outlined as follows.

### 1. Necroptosis May Just Be a Way of Killing Cancer Cells and Removing Them

The simplest explanation would be that since resistance to cell death is one of the hallmarks of a cancer cell (Hanahan and Weinberg, 2011), cancer cells are selected to become resistant to necroptosis, and one of the easiest ways in which expression of such an essential protein can be regulated is through methylation of its genomic DNA, thus silencing its transcription. However, in this case, cancer cells must still have a necroptotic signal that is somehow activated upon becoming a cancer cell. For necroptosis, we are unaware of any specific stress, save immunological stress (see below) in which becoming a cancer cell would activate the necroptosis pathway. It is highly possible that NK cells or cytotoxic T lymphocytes could target cancer cells through a Fas ligand or TRAIL-mediated necroptosis in response to a lack of MHC1 molecules (for NK cells), which sometimes happens in cancer cells or through the presentation of a cancer-specific antigen (cytotoxic T cells). Likewise, TNF-α is a very common cytokine secreted by many immune cells and is often released to target macrophages to an inflammatory area.

### 2. RIPK3 Could Alter Metabolism for the Cancer Cell

RIPK3 activities are due to its enzyme function as a kinase for MLKL. However, MLKL is definitely not the only substrate for RIPK3. RIPK3 phosphorylates PYGL, GLUL, GLUD1, and other metabolic enzymes mainly associated with mitochondrial metabolic pathways to increase aerobic, which may or may not be solely associated with necroptosis (Yang et al., 2018, Zhang et al., 2009). In this case, it would then follow that too much mitochondrial metabolism might inhibit the Warburg effect whereby cancer cells rely on glycolysis to produce anabolic intermediates, which would not be advantageous to a cancer cell and, therefore, RIPK3 would be selected against.

### 3. RIPK3 Repression Could Be Associated With Cell Cycle Progression

Deletion of RIPK3 was shown to drive reprograming of MEFs into pluripotent stem cells (Al-Moujahed et al., 2019). While this idea would be an attractive hypothesis, given that cancer cells often maintain a “stem like” phenotype associated with epithelial-to-mesenchymal transition (EMT), the data of Al-Moujahed et al. (2019) led them to conclude that this was because RIPK3 affects the expression of cell cycle/cell division genes. RIPK3 KO MEFs had a growth rate that was significantly lower than that of WT MEFs and phosphoproteomic analysis of other RIPK3 phosphorylated peptides indicated that many of them were associated with the cell cycle (Al-Moujahed et al., 2019, Wu et al., 2012). Little is known about cell cycle-specific substrates; it is likely that RIPK3 has other functions of its kinase activity outside of necroptosis. However, the cell cycle would be reduced, which is not often advantageous in cancer other than becoming more mesenchymal-like. Likewise, this hypothesis would not explain the favorable prognostic information that was correlated with MLKL expression, unless MLKL was somehow also involved.

### 4. Necroptosis May Be Important for Immune Surveillance of Cancer Cells

**Fig. 2:**
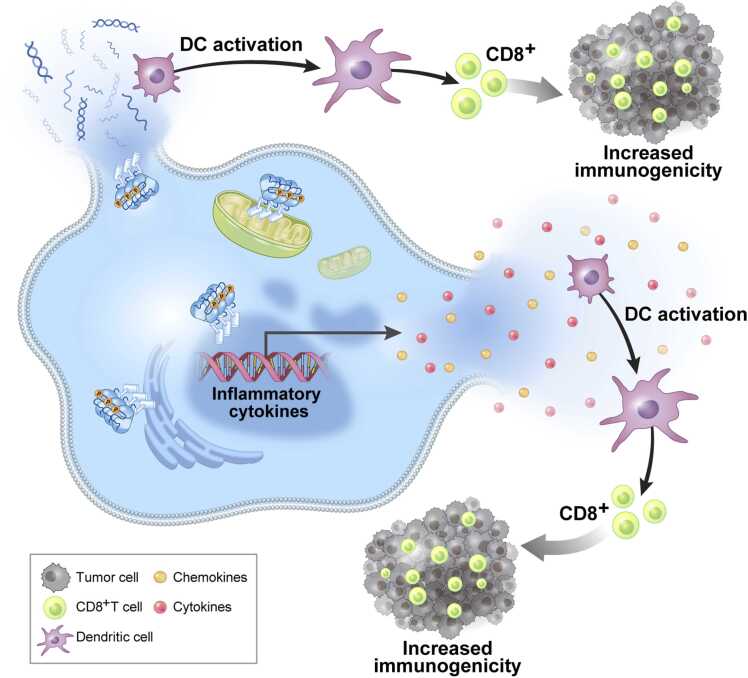
Release of damage-associated molecular patterns through necroptosis activation induces robust antitumoral immunity. Induction of cell death through necroptosis promotes the release of damage-associated molecular patterns, activating immune responses, and modulating the tumor microenvironment.

This may be the most important reason for RIPK3 silencing in cancer. We now know that necroptotic cells may play several roles in both innate immunity and in molding subsequent adaptive immunity. This is thought to occur through the release of danger-signaling molecules known as damage-associated molecular patterns (DAMPs) (Choi et al., 2019, Kaczmarek et al., 2013), which then activate PRRs of innate immune cells to upregulate phagocytosis and the responses to pathogens and potentially neoplastic cells. Since RIPK3 could be potentially involved in both the DAMP release and some of the signals generated by PRRs, this would make a lot of sense for its expression to be selected against. Dying necroptotic cells continue the *de novo* synthesis of cytokine and chemokine cells (Gong et al., 2017a, Orozco et al., 2019, Zhu et al., 2018), likely due in part to inflammatory signals that are known to activate necroptosis. Activation of RIPK1/RIPK3 has been shown in many studies to upregulate inflammatory cytokines and chemokines that help to promote cross-priming of CD8^+^ T cells (Park et al., 2021, Ren et al., 2017, Snyder et al., 2019, Yatim et al., 2015) and/or release DAMPs (Aaes et al., 2016); thus, necroptosis is believed to upregulate efficient immunogenic anticancer responses (Aaes et al., 2016, Liang et al., 2024, Mannion et al., 2024, Park et al., 2021, Rucker et al., 2024, Snyder et al., 2019, Yatim et al., 2015) (Fig. 2). ZBP-mediated necroptosis, for instance, is reported to reverse ICB unresponsiveness in mouse models of melanoma immune checkpoint blockade (ICB)-based monotherapies. However, necroptosis has also been observed to have opposite effects on ICB-based monotherapies in non–small-cell lung cancer (Duangthim et al., 2024). Deficiency of RIPK3 in hepatocellular carcinoma is reported to increase M2 tumor-associated macrophages population associated with antitumor immunity, also leading to the conclusion that RIPK3 suppresses the protumorigenic effects (Wu et al., 2020) of the immune system in favor of the antitumor immunogenic responses.

### 5. Necroptosis Can Be Activated by Chemotherapy

In our previous work, we found that necroptosis was induced by diverse chemotherapeutics when RIPK3 was present (Koo et al., 2015) and that cancer cells not only responded by inducing necroptosis, but the amount of total cell death was increased relative to cells that did not express RIPK3, suggesting that necroptosis could play a role in chemotherapy resistance in cancers via cell killing. Since most of the prognostic information from the previous studies was in patients that may have received chemotherapy (Colbert et al., 2013, Conev et al., 2019, Ertao et al., 2016, Feng et al., 2015, Koo et al., 2015, Lin et al., 2023, Nicole et al., 2022), it would logically follow that patients with high RIPK3 levels would respond better to killing cancer cells, and also, as mentioned in the fourth hypothesis above, contributing to immunogenic cell death.

While there are several nonmutually exclusive hypotheses as to why and how RIPK3 would provide anticancer effects, the evidence for pro-cancer effects at this point largely seems to point to a single main explanation: RIPK3 signaling (sometimes coupled with limited necroptosis itself) leading to inflammation, which contributes to an inflammatory and immunosuppressive tumor microenvironment (Jiao et al., 2018, Mohammed et al., 2023, Seifert et al., 2016, Strilic et al., 2016, Vucur et al., 2023, Wang et al., 2018), which leads to a situational outcome completely opposite of the one proposed in the fourth hypothesis above.

So how does one explain the possible contradiction in the data suggesting opposing effects of necroptosis on cancer, especially with regard to the different outcomes following immune cell activation? Of course, much may be explained by the obvious differences in the cancer type and cellular systems used. One report, however, suggests that it may have less to do with overall cellular context so much as it is the amount of RIPK3 and the extent of the necroptotic signaling, as well as the duration of the signaling. Thus, they have proposed that, at least in hepatocarcinogenesis, sublethal necroptosis occurs where limited but chronic necroptosis is activated, which contributes to immune cell activation that is immunosuppressive (Vucur et al., 2023), while lethal necroptosis leads to an anticancer effect. Others have also argued that chronic necroptosis leads to pro-cancer inflammation, while high-intensity acute necroptosis triggers immunogenic responses (Yan et al., 2022). Interestingly, in the Vucur et al. (2023) report, the difference between whether lethal and sublethal necroptosis occurs is whether NF-κB is activated significantly, with repression of its activity leading to lethality. This is curious because NF-κB is argued to be essential in immunogenic necroptosis that leads to anticancer responses (Yatim et al., 2015), though this has been disputed by others (Ren et al., 2017). Nevertheless, Vucur et al.’s (2023) report is consistent with other data showing that necroptosis in the absence of NF-κB leads to antitumor immunity (Rucker et al., 2024).

Survival of cells undergoing necroptosis has been demonstrated in situations where the necroptotic activation of MLKL is limited and cells continually repair and shed plasma membrane bubbles, due to ESCRT action (Gong et al., 2017b). Importantly, various components of the ESCRT machinery are upregulated by HIF-1α and HIF-2α (Gong et al., 2019), and therefore upregulated during hypoxia, which is well-known to be a pro-cancer stimulus. In the context of ESCRT activity, shed bubbles may act as signal-transducing messengers (Gong et al., 2019). Considering that it is believed that DAMPs and cytokines are responsible for both immunological effects (pro- and anticancer). Might the differences between the effects of lethal and sublethal necroptosis on cancer be explained not only by differences in what cytokines and DAMPs are released and the quantity released but also by how it is released (ie, direct leakage from necroptotic cells vs shedding bubbles containing membrane-bound proteins and also enclosed soluble ones)? This idea has yet to be examined, but could provide one potential explanation for pro-cancer immunomodulators and anticancer ones. Many other explanations are, of course, possible at this point.

## CONCLUSION

Understanding the roles of RIPK3 and necroptosis is critical to being able to modulate possible therapeutics for cancer and inflammatory diseases. RIPK3 plays important roles in cancer and likely cancer therapy, in part by killing cancer cells, repressing their growth, and/or promoting efficient anticancer responses by the immune system.

## FUNDING AND SUPPORT

This work was supported by the National Research Foundation of Korea to YSK (grant number 2022R1A2B5B03001824) and by the Korea Health Technology R&D Project through the Korea Health Industry Development Institute, funded by the Ministry of Health and Welfare, Republic of Korea (grant number: HR21C1003). MJM is currently supported by a Research Project Investigator award from the U.S. National Institutes of Health Via the Oklahoma IDeA Network of Biomedical Research Excellence program.

## CRediT Authorship Contribution Statement

**Michael J. Morgan** and **You-Sun Kim:** Writing – original draft, conceptualization.

## DECLARATION OF COMPETING INTERESTS

The authors have no affiliations, financial involvement, or interests in any organization or entity with a direct or indirect stake in the subject matter discussed in this manuscript.
